# Review of Aging Evaluation Methods for Silicone Rubber Composite Insulators

**DOI:** 10.3390/polym15051141

**Published:** 2023-02-24

**Authors:** Zhou Zeng, Pan Guo, Ruoshuang Zhang, Zhirui Zhao, Jiankang Bao, Qian Wang, Zheng Xu

**Affiliations:** 1 College of Physics and Electronic Engineering, Chongqing Normal University, Chongqing 401331, China; 2 State Grid Chongqing Electric Power Company, Electric Power Research Institute, Chongqing 401121, China; 3 School of Electrical Engineering, Chongqing University, Chongqing 400044, China

**Keywords:** silicone rubber insulating material, aging mechanism, evaluation method, applicability, effectiveness

## Abstract

Silicone rubber insulation material is widely used for the external insulation of power systems. During the continuous service of a power grid, it will be seriously aged due to the influence of high voltage electric fields and harsh climate environments, which will reduce its insulation performance and service life and cause transmission line failure. How to evaluate the aging performance of silicone rubber insulation materials scientifically and accurately is a hot and difficult issue in the industry. Starting from the composite insulator, which is the most widely used insulating device of silicone rubber insulation materials, this paper expounds the aging mechanism of silicone rubber materials, analyzes the applicability and effectiveness of various existing aging tests and evaluation methods, especially discusses the magnetic resonance detection methods emerging in recent years, and finally summarizes the characterization and evaluation technology of the aging state of silicone rubber insulation materials.

## 1. Introduction

Insulators can be classified into ceramic insulators, glass insulators, and composite insulators according to the materials. Insulators can fix and suspend transmission conductors and also meet the insulation requirements of specific voltage levels. Insulators have been used in the field of external insulation of power systems for more than 120 years. In the early stage, ceramic insulators and glass insulators were mainly employed. With the improvement in voltage level, the mechanical load borne by insulators is increasing, and the defects of ceramic and glass insulators are gradually highlighted. In terms of performance, ceramic and glass insulators are easily damaged during transportation, loading, and unloading, resulting in flashover in a polluted environment. During the service, the presence of separated water droplets concentrates the field strength at the junction of the silicone rubber, air, and water droplets, resulting in strong partial discharge around the water droplets before flashover. Partial discharge causes physical and chemical changes on the surface of silicone rubber, destroys the molecular structure of silicone rubber, and produces many factors that are not conducive to hydrophobicity, leading to the decline in hydrophobicity. Economically, the production and delivery cycle of ceramic and glass insulators is long, and the handling and transportation costs are high.

**Figure 1:**
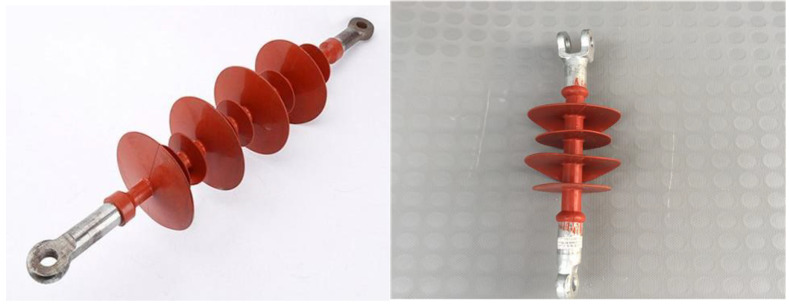
High-temperature vulcanized silicone rubber composite insulator.

High-temperature vulcanized silicone rubber (HTV silicone rubber) material [1,2,3] originated in the United States, Germany, France, the former Soviet Union, and other countries. In the 1970s, the HTV silicone rubber composite insulator (Figure 1) was first introduced in Germany. High-temperature vulcanized silicone rubber is the material used for the umbrella skirt sheath of composite insulators. The material combines elements in the molecule through covalent bonds, and the bonding force is relatively weak. The silicone rubber insulating umbrella skirt of the composite insulator is a kind of polymer, which is a component of the external insulation of the product. It is made of mixed insulating rubber through a special mold and is vulcanized under the pressure of rubber vulcanization. A composite insulator is the insulating part of the suspended conductor on the transmission line. During the operation, the insulation performance of the umbrella skirt is extremely vulnerable to the influence of the surrounding environmental factors, especially the influence of the pollutants, which will lead to the pollution flashover trip and power failure of the transmission line. In order to ensure the safe operation of the transmission line, in addition to ensuring the surface insulation performance by increasing the creepage distance by the number of umbrella skirts, we must also maximize the hydrophobicity and hydrophobicity migration performance of the silicone rubber material in the umbrella skirt insulation to improve the pollution flashover voltage level on the insulator surface. In the long-term operation, due to the comprehensive influence of light, ultraviolet, strong current, humidity, pollution, corona discharge, flashover, and other factors, there are more serious pulverization, hardening, silver grain, and aging (irreversible physical and chemical property changes) phenomena such as poor hydrophobicity and sewage resistance. The insulation performance and operating life are reduced, resulting in transmission line accidents that seriously affect the safe and stable operation of the power system. During use, due to the combined effect of many factors such as high-voltage electric fields, high-temperature sunshine, harsh climatic environments, and pollution, the umbrella skirt of the composite insulators gradually ages over time [4,5,6,7]. Rainfall is an important factor affecting the pollution accumulation of insulators. The upper surface of insulators is wetted and washed by rain, resulting in the loss of salt and ash. It is difficult for rain to wash the lower surface, so it is easy for the lower surface to accumulate serious pollution often. The scouring effect of rainfall is related to the scouring angle and rainfall intensity. With the increase in the scouring angle, the area of insulators to be cleaned will increase. The greater the rainfall intensity, the more pollution will be lost. The aging of the insulators will show the degradation of hydrophobicity [8]. The degradation of hydrophobicity [9] will seriously affect the insulation performance of composite insulators and increase the probability of flashover accidents. In 2007, the State Grid Corporation of China [10] found that the annual damage rate of composite insulators was about 0.005%. According to the investigation results of the International Council on Large Electric Systems (CIGRE) and the Institute of Electrical and Electronic Engineers (IEEE) on the failure of composite insulators in operation, the aging of the silicone rubber insulation skirts of composite insulators accounted for 64% of the failures [2]. Therefore, it is of great value to research the aging laws and influencing factors of high-temperature vulcanized silicone rubber insulation materials [11]. How to use scientific and accurate means to realize the correct evaluation of the aging of composite insulators is a problem worth studying.

In this paper, the aging mechanism of high-temperature vulcanized silicone rubber materials is briefly introduced, and the aging evaluation methods of silicone rubber insulating aprons are analyzed in detail, including macroscopic and microscopic methods, as well as the magnetic resonance measurement method that combines macroscopic and microscopic methods developed in recent years; the advantages, disadvantages, and application scope of each detection method are discussed in order to provide a reference for the quantitative evaluation of the aging of the composite insulator shed.

## 2. Aging Mechanism

**Figure 2:**
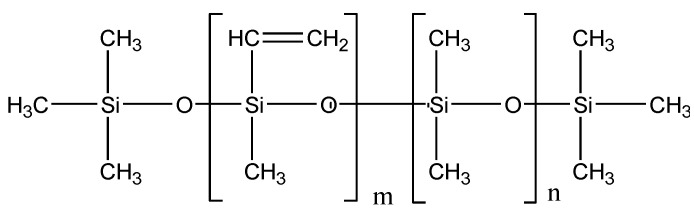
The formula of poly dimethyl siloxane.

A composite insulator apron is mainly composed of high-temperature vulcanized silicone rubber raw material, white carbon black, aluminum hydroxide, flame retardant, colorant, and a release agent, while silicone rubber is an elastomer material, which is mainly cross-linked by linear Polydimethylsiloxane (PDMS). The molecular formula of PDMS is shown in Figure 2. It can be seen that PDMS is a copolymer of a methyl vinyl siloxane chain link and dimethyl siloxane chain link. The nonpolar methyl group with symmetrical side chains shields the polarity of the main chain siloxane bond, and the interaction with polar water molecules is weak. Macroscopically, the silicone rubber umbrella skirt cannot be soaked by water, which makes the silicone rubber surface show excellent hydrophobicity. At the same time, the silicone rubber umbrella skirt has a certain hydrophobicity mobility: there are a small number of small molecules mainly composed of low molecular siloxanes in the umbrella skirt, and these small molecules diffuse from the body to the surface, causing the dirty surface to be wrapped by small molecules so that the dirt also shows hydrophobicity.

The atoms in PDMS molecules are bound by strong covalent bonds, while the intermolecular force is weak. In essence, the aging of the composite insulator apron is a manifestation of the changes in the microstructure of silicone rubber insulation materials under the effects of pollution, corona arc discharge, ultraviolet radiation, moisture, temperature changes, and chemical factors. It is mainly manifested in the degradation reaction caused by the fracture of the Si-C bond and C-H bond in PDMS; the side chain methyl is especially easy to fracture and is accompanied by the cross-linking reaction generated by the ionic polymerization after the fracture [12,13]. A dense inorganic silicon oxide layer is formed on the surface of the aged silicone rubber umbrella skirt. Although the inorganic silicon oxide layer can retard the further aging of the umbrella skirt in a certain layer degree, it is easy to form broken surface micropores after being damaged by external stress. The micropores provide a channel for the diffusion of small molecules, restoring the hydrophobicity of the silicone rubber umbrella skirt surface that has lost hydrophobicity, so as to prevent water molecules from diffusing into the silicone rubber insulator through the microporous surface on a certain layer. However, the small molecules diffused to the surface will reach the flash point and volatilize at a high temperature, which will destroy the hydrophobicity of the apron surface again, and the small molecules in the body will diffuse to the surface again under the effect of poor surface energy. The above process continues to circulate, and finally, the content of small molecules in the silicone rubber continues to decrease. When the microholes on the surface of the umbrella skirt develop into macroholes, the water invades the inside of the umbrella skirt, which makes it easy to form a conductive path, resulting in the aging of the inside of the umbrella skirt and the decline in the insulation performance. Finally, the umbrella skirt is withdrawn from service [14].

## 3. Test Methods

### 3.1. Macroinspection Method for Aging of Composite Insulators

At present, the macroscopic methods for testing the aging state of silicone rubber insulated umbrella skirts are mainly as follows:

#### 3.1.1. Appearance Inspection

Observe the state of the silicone rubber umbrella skirt by observing the equipment on the ground or climbing the tower to determine whether there is damage, a crack, an electric breakdown, and other phenomena. However, this method relies on the subjective judgment of the inspector, and it is difficult to find internal faults. It is mainly used to check large surface damage.

#### 3.1.2. Flashover Voltage Detection and Electric Field Distribution Test

Due to the difference in the operating environment, the aging degree of the silicone rubber composite insulator has a large dispersion, so the pollution flashover voltage and lightning flashover voltage of the insulator has no obvious relationship with the operating time [8]. Cheng Yangchun et al. [9] measured the electric field distribution of different insulators and the influence of pollution on the electric field distribution. The aging results of the umbrella skirt obtained by this method were basically consistent with the infrared thermal imaging, but it was only effective for the fault of the fully wetted composite insulators, and the detection cost was high.

#### 3.1.3. Hydrophobicity Test

The hydrophobicity test mainly includes the hydrophobicity level (HC) test and water contact angle test. Amin et al. [12] used a multifactor aging simulation method to age the silicone rubber umbrella skirt and perform a hydrophobicity grading test. The results showed that the HC grade of the umbrella skirt increased with the increase in the aging degree. Although this method can truly reflect the hydrophobicity of the silicone rubber umbrella skirt, it has strong subjectivity, large dispersion, and more investment in safety assurance [13]. Wang Fochi et al. [14] tested the water contact angle of silicone rubber umbrella skirts with different aging degrees, and the results showed that the water contact angle of the umbrella skirts decreased with the increase in the aging degrees. The precision of this method is very high, but the test conditions are harsh, which can only be completed in the laboratory using plane materials, and it is difficult to detect in the field.

#### 3.1.4. Leakage Current

The current flowing when the dirt layer on the surface of the umbrella skirt is damp is the leakage current. Since too much dirt on the surface of the umbrella skirt is likely to cause flashover accidents, the leakage current can reflect the operation of the silicone rubber umbrella skirt [15,16]. However, the leakage current is not only related to the pollution on the umbrella skirt surface but also related to the operating voltage, environmental humidity, temperature, etc., so the measurement accuracy of the leakage current method is vulnerable to environmental factors [17,18].

#### 3.1.5. Ultrasonic Testing

Use the characteristics of ultrasonic reflection, refraction, and mode transformation at the crack of the umbrella skirt to judge the defect of the insulator. Xie Congzhen et al. [19] used different detection and media to detect silicone rubber insulators and found that the detection effect of radio frequency waves is superior to other detection methods. Wang H [20] established a cylinder adhesion contact model to describe the obvious distortion of ultrasonic propagation caused by the polymer interface kissing defect of composite insulators. By measuring the ultrasonic nonlinear parameters of each kissing defect and complete sample, the 0.7 existing in the composite insulator was successfully identified as the μ M Kissing defect. However, the judgment of ultrasonic testing on the type, size, and location of the defects is highly dependent on the experience of operators and can only be detected when cracks occur.

#### 3.1.6. Optical Detection

Mainly performed by infrared imaging. Li Zhenyu et al. [21] used infrared thermal imaging technology to monitor the temperature characteristics of different external insulating materials and conveniently obtained the temperature change characteristics of the material surface. However, this detection technology is vulnerable to the interference of ground radiation in the daytime, the detection risk coefficient at night is high, and the measurement results are vulnerable to the impact of environment and pollution, and the thermal fault is obvious only when the partial discharge is significant.

#### 3.1.7. Mechanical Test

Xie Siyang et al. [22] carried out tests on the mechanical properties and damage characteristics of umbrella skirts of composite insulators. The tensile strength and elongation at the break of umbrella skirts were measured by an electronic universal testing machine, and the mechanical property characteristics and change rules of the umbrella skirts of insulators operating in different years were analyzed. This method found that there was a clear boundary between the mechanical properties of damaged and undamaged umbrella skirts, but it was not sensitive enough to detect the early aging state of composite insulators.

In general, the aging performance of a silicone rubber umbrella skirt can only show a difference after obvious deterioration by means of macroscopic electrical, mechanical, and other means, and qualitative testing is the main method. In fact, after the silicon rubber umbrella skirt is deteriorated due to the influence of the electric fields and mechanical and environmental factors during operation, the early aging process may be relatively slow. At this time, the electrical, mechanical, and other macroproperties of the umbrella skirt may not have significantly decreased, but the microstructure of the umbrella skirt has changed. Once it develops to a certain stage, the aging will significantly accelerate, and in serious cases, it will lead to failures, broken strings, and other accidents. Therefore, it is necessary to study and establish the aging characterization method of the microstructure of the silicone rubber insulating umbrella skirt.

### 3.2. Microinspection Method for Aging of Composite Insulators

#### 3.2.1. Thermal Weight-Loss Analysis

The silicone rubber apron is thermally decomposed in the heating process, which leads to the change in the material quality. The thermogravimetric curve can be obtained by recording the mass of the material at each temperature point with the thermobalance, realizing the quantitative detection of the content of the main components in the silicone rubber apron, so as to judge the aging of the apron. However, thermogravimetric analysis is a destructive method.

#### 3.2.2. Thermal Stimulation Current

By measuring the short-circuit current characteristic curve of the external circuit of the insulator under the set temperature and electric field, the trap parameters of the umbrella skirt are analyzed [23,24]. However, the thermal stimulation current is a destructive detection method, and the detection is vulnerable to environmental factors, so it cannot be used for engineering site detection.

#### 3.2.3. Infrared Spectrum

**Figure 3:**
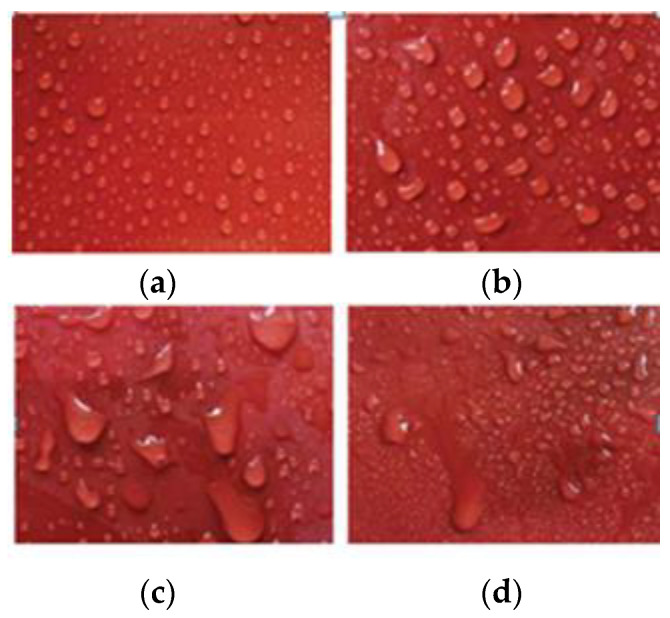
Hydrophobicity grading diagram. (**a**) HC1; (**b**) HC2; (**c**) HC3; (**d**) HC4.

**Figure 4:**
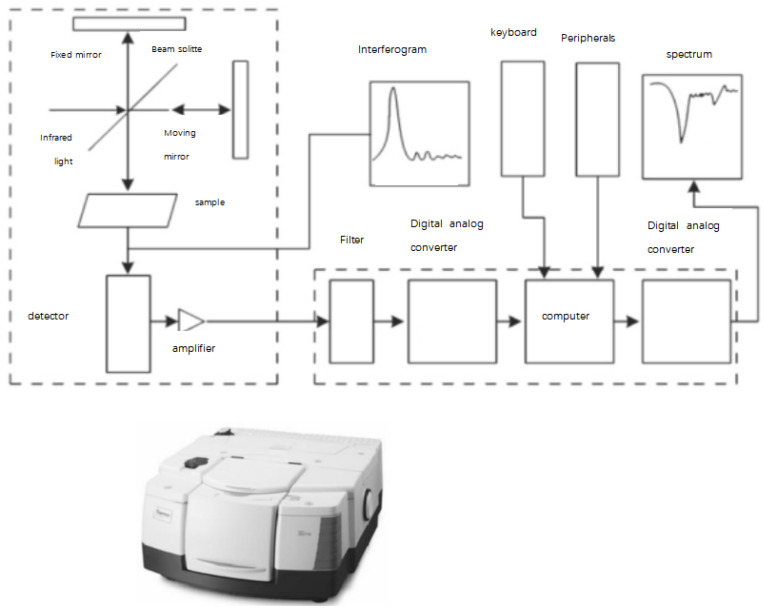
Measuring principle of Fourier infrared spectroscopy.

Kuang Fan et al. [25] used a Fourier infrared spectrometer to test the aging degree of composite insulators running at the same voltage level with different service lives, as shown in Figure 3 and Figure 4. The chemical bond in the material is determined by the peak value corresponding to the wavelength of different functional groups. The FTIR absorption peak strength of the outer layer of the sample is taken as the characteristic quantity reflecting the aging state of the composite insulator and is compared with the measurement results of the nuclear magnetic resonance method. The results showed that the surface area of the composite insulator was seriously polluted, its absorption peak intensity was lower than that of the inner layer, the breaking degree of the organic side chain was more serious than that of the inorganic main chain, the decreasing percentage of the absorption peak was greater, and the absorption peak strength of the insulator decreased with the increase in the service life. However, traditional Fourier infrared spectroscopy can only evaluate the overall aging of the surface and cannot reflect the longitudinal depth aging information. Microinfrared spectroscopy can focus the high-throughput interference infrared beam on the small area of the sample for analysis with high accuracy and provide the molecular structure, functional group information, and spatial distribution of functional group content at each point in the spatial position. However, the absorption peak intensity is not stable enough. The spectrum analysis is mainly based on experience, and the equipment is expensive and cumbersome, which is not convenient for online detection on the project site.

#### 3.2.4. Slow Positron Beam

The positron defects (such as the free volume in the inorganic layer) capture annihilation characteristics to reflect the local characteristics of defects (such as the electron density, momentum distribution, and other information of the defect location), and the positron is annihilated and released by detecting the positron in the material γ. The information carried by photons is used to obtain the microstructure information of the positron annihilation position. Xiang et al. [26,27] studied the aging process of butyl rubber under a 100 °C air atmosphere by using positron annihilation lifetime spectroscopy. The results showed that the free volume and concentration of PBR decreased with the increase in aging time.

#### 3.2.5. Terahertz Detection

Zhang Xuemin et al. [28] used the Terahertz vector network analyzer to conduct experimental tests on insulators, established a composite insulator aging detection model based on the terahertz signal transmission characteristics, and used a small terahertz sensor to conduct on-site tests. The results showed that the aging degree of the insulators was proportional to the return loss parameters, and it is feasible to use the THz wave to detect the aging degree of composite insulators without contact. The shortage of the experiment is that the signal is vulnerable to the interference of the surrounding environment, and the echo waveform fluctuates greatly, so effective detection requires a lot of data support.

In general, microscopic detection methods of silicone rubber insulation materials, including other commonly used technologies such as gas chromatography–mass spectrometry (GC-MS), can effectively characterize the microscopic change processes such as polymer phase transition, interface characteristics, and the physical aging of polymers from the level of molecular chain segment movement, and can help us deeply understand the aging process of silicone rubber composite insulators from a microscopic perspective. However, the equipment is usually heavy, expensive, and requires a high testing environment. It is generally used for offline research under laboratory conditions and cannot be used for in situ online quantitative testing of silicone rubber insulators.

#### 3.2.6. Magnetic Resonance Testing Method for Aging of Composite Insulators

To sum up, the ideal testing method should be able to analyze the aging status of silicone rubber from the microlevel, and the measurement process is simple and fast, the equipment cost is low, and the detection is portable and safe. It can realize the in situ nondestructive testing and quantitative evaluation of the aging degree of the silicone rubber umbrella skirt and become a widely used engineering measurement method.

Nuclear magnetic resonance technology is fast and convenient to measure and can realize quantitative nondestructive testing. In materials science, it has been used to measure the crosslinking density of polymer materials, control the quality of the rubber production process, detect the aging of rubber and polymer materials, and measure the content of each component in polymers [29,30]. The main chemical bonds in the molecules of silicone rubber insulating aprons are Si-O, Si-C, and C-H bonds. In the aging process of the apron, under the combined action of ultraviolet radiation, partial discharge and other factors, chemical bonds such as Si-C and C-H break, and the cracking reaction forms free radicals -H, -CH_3_, and short chains containing active radicals such as -O, -Si, and -CH_2_. The free radicals and short chains containing active radicals will recombine with each other to form relatively stable chemical bonds, that is, the cross-linking reaction occurs. In the process of short-chain cross-linking, longer main chains, double main chains, and main chains with side chains will be formed, which will increase the cross-linking density of the silicone rubber materials and eventually lead to the change in the chemical state of atoms such as H and C and their groups. Therefore, the aging state of the umbrella skirt can be reflected by measuring the atomic chemical structure information contained in its NMR echo signal.

A. E. Somers [31] quantified the aging of silicone rubber with nuclear magnetic resonance technology and compared the natural silicone rubber with different aging degrees with the transverse relaxation time T_2_ and nuclear magnetic resonance 13C spectra of the H nucleus. The experimental results showed that with the increase in the aging degree of silicone rubber, the transverse relaxation time T_2_ of the H core decreased, the 13C spectrum broadened, and the molecular mobility decreased. At the same time, it is pointed out that silicone rubber is a multicomponent material, and its T_2_ spectrum has more than one peak. E. Somers et al. used the overall transverse relaxation time T_2eff_ parameter to quantify the aging degree of silicone rubber. In order to realize the field measurement, nuclear magnetic resonance instruments need to be portable. People often use low-field permanent magnetic nuclear magnetic resonance instruments (usually B0 is less than 0.3T) to measure the magnetic resonance signal of the material H core [32].

**Figure 5:**
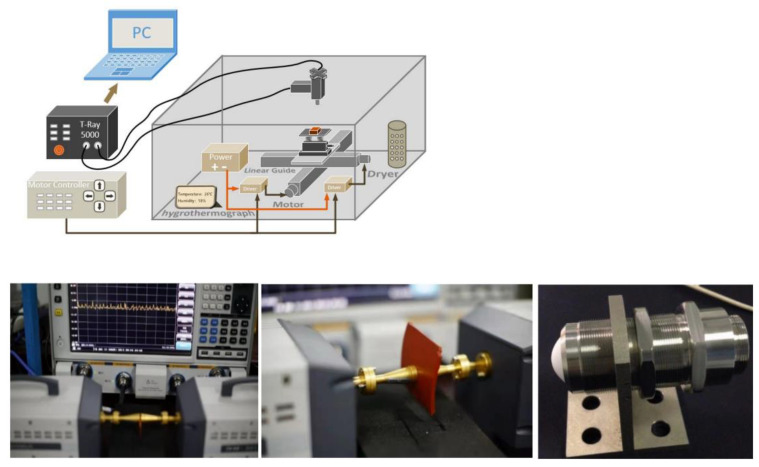
Terahertz detection experimental platform.

A. Guthausen et al. [33] found that the increase in T_2_ corresponds to the enhancement in intermolecular mobility. Halmen N et al. [34] studied the feasibility of monitoring the process of determining the degree of crosslinking and curing with single-sided nuclear magnetic resonance in a nondestructive manner, as shown in Figure 5. The experimental results showed that a single-sided nuclear magnetic resonance measurement [35,36,37] can distinguish cross-linked polyethylene samples with different cross-linking degrees and can also monitor the homogeneity of the samples and the curing kinetics of adhesives. The experimental results were in good agreement with other reference tests (wet chemical analysis, differential scanning calorimetry, dielectric analysis). In addition, the influence of the sample temperature on the characteristic relaxation time could also be observed.

**Figure 6:**
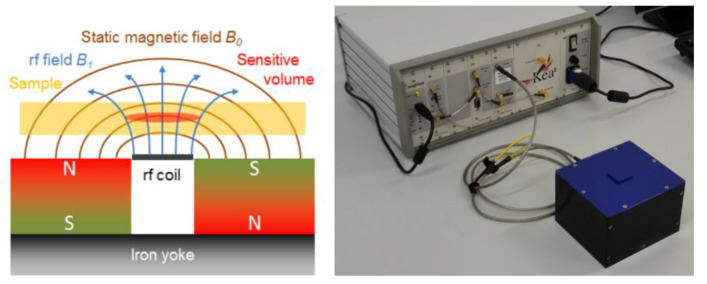
Schematic diagram of unilateral nuclear magnetic resonance system measurement.

**Figure 7:**
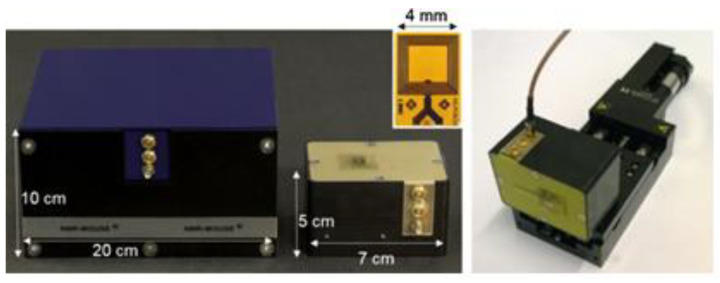
Miniaturized NMR MOUSE sensor.

**Figure 8:**
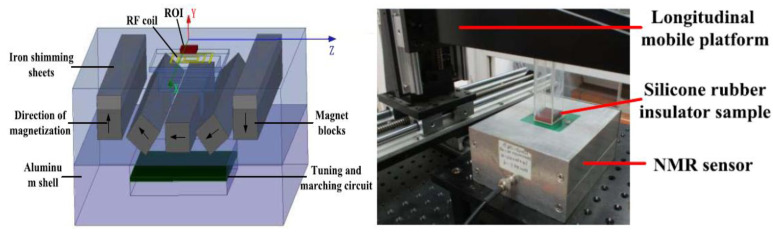
Schematic diagram of portable single-sided magnetic resonance sensing measurement of arc permanent magnet structure.

In order to realize the online detection of the umbrella skirt of the silicone rubber composite insulator, a structure-matching magnetic resonance sensor must be designed to meet the requirements of nondestructive testing. In order to realize the nondestructive testing of samples, researchers learned from the “inside out” concept of nuclear magnetic resonance logging technology [38], that is, the sensor is placed in the well cavity to measure the liquid around the well cavity. This concept subverts the traditional idea of placing samples in magnets for measurement and then successively designing inside-out magnetic resonance sensors with different structures. The most representative is the portable single-sided magnetic resonance sensor NMR-MOUSE [39] designed by Bl ü Michael of the Aachen University of Technology in Germany. As shown in Figure 6, the measurement sample is located on one side of the permanent magnet, and the measurement area is a gradient magnetic field [40,41]. The weight of NMR-MOUSE is less than 2 kg, which has good openness and portability and can be used for the nondestructive testing of food, cultural murals, rubber, and polymer materials. Drawing on the ideas of NMR MOUSE, the author’s team [42,43] designed a portable single-sided magnetic resonance sensor (68 mm) with an arc permanent magnet structure in 2016 (68 mm × 129 mm × 154 mm, 3 kg), see Figure 7 and Figure 8. Its measuring area is an ultrathin layer (10 mm × 10 mm × 759.1 μm). In combination with the commercial measuring circuit system, it can be measured closely on the surface of the silicone rubber umbrella skirt. Based on the designed arc magnetic resonance sensor, while keeping the working frequency of the sensor unchanged, the high-precision three-dimensional stepping motor is used to adjust the distance between the silicone rubber umbrella skirt and the sensor, and the transverse relaxation time T2 of the silicone rubber umbrella skirt at different longitudinal depths is measured. The measurement results showed that with the increase in the measurement depth, the transverse relaxation time T2 of NMR gradually increased and finally tended to be stable, indicating that with the increase in the depth, the aging degree of the silicone rubber umbrella skirt gradually weakened, which is the same trend as the measurement results of the microinfrared imaging system in the laboratory environment [2].

## 4. Conclusions

The macroscopic means can only detect the obvious deterioration of the aging performance of composite insulators, and qualitative detection is the main method. Micro means can reflect early aging, but most of the equipment is expensive and the experimental environment is harsh. As a means to detect the microstructure of materials, nuclear magnetic resonance (NMR), analogous to the current medical NMR technology, is beneficial to all of mankind. If the portable in situ quantitative nondestructive testing of the aging degree of the silicone rubber umbrella skirt in the power system can be realized and its timely replacement can be guided, it will not only ensure the safe operation of the power system but will also reduce unnecessary expenses. It has great economic value and can provide a new idea for the research on the insulation aging of power equipment, and it will certainly be welcomed by the power system. However, the current portable single-sided nuclear magnetic resonance sensor still has the following difficulties in detecting the silicone rubber umbrella skirt: (1) The measurement results will be interfered with by temperature changes. (2) The signal of the low-field single-sided nuclear magnetic resonance sensor itself is weak, and the electromagnetic wave interference in the project site further leads to the low signal-to-noise ratio of the measurement signal, which affects the signal analysis. (3) The existing nuclear magnetic resonance testing studies only quantitatively characterized the hydrophobicity of the silicone rubber umbrella skirt based on the transverse relaxation time T2 but did not quantitatively characterize the influence of its hydrophobicity recovery ability on the aging degree, which made it impossible to make a reasonable assessment of the service life of the silicone rubber umbrella skirt.

## Data Availability

The relevant data of this article can be found on CNKI (https://www.cnki.net/ (accessed on 1 January 2023)). There is no conflict of interest.
